# Contact diaries versus wearable proximity sensors in measuring contact patterns at a conference: method comparison and participants’ attitudes

**DOI:** 10.1186/s12879-016-1676-y

**Published:** 2016-07-22

**Authors:** Timo Smieszek, Stefanie Castell, Alain Barrat, Ciro Cattuto, Peter J. White, Gérard Krause

**Affiliations:** NIHR Health Protection Research Unit in Modelling Methodology and MRC Outbreak Centre for Outbreak Analysis and Modelling, Department of Infectious Disease Epidemiology, School of Public Health, Imperial College London, London, UK; Modelling and Economics Unit, Centre for Infectious Disease Surveillance and Control, Public Health England, London, UK; Department for Epidemiology, Helmholtz-Centre for Infection Research, Braunschweig, Germany; Aix Marseille Université, Université de Toulon, CNRS, CPT, UMR 7332, Marseille, 13288 France; Data Science Laboratory, ISI Foundation, Torino, Italy; Hannover Medical School, Hannover, Germany

**Keywords:** RFID, Proximity sensor, Contact network, Contact diary, Measurement error, Acceptability, Infectious disease, Network epidemiology, Network model, Infection transmission

## Abstract

**Background:**

Studies measuring contact networks have helped to improve our understanding of infectious disease transmission. However, several methodological issues are still unresolved, such as which method of contact measurement is the most valid. Further, complete network analysis requires data from most, ideally all, members of a network and, to achieve this, acceptance of the measurement method. We aimed at investigating measurement error by comparing two methods of contact measurement – paper diaries vs. wearable proximity sensors – that were applied concurrently to the same population, and we measured acceptability.

**Methods:**

We investigated the contact network of one day of an epidemiology conference in September 2014. Seventy-six participants wore proximity sensors throughout the day while concurrently recording their contacts with other study participants in a paper-diary; they also reported on method acceptability.

**Results:**

There were 329 contact reports in the paper diaries, corresponding to 199 contacts, of which 130 were noted by both parties. The sensors recorded 316 contacts, which would have resulted in 632 contact reports if there had been perfect concordance in recording. We estimated the probabilities that a contact was reported in a diary as: *P* = 72 % for <5 min contact duration (significantly lower than the following, *p* < 0.05), *P* = 86 % for 5-15 min, *P* = 89 % for 15-60 min, and *P* = 94 % for >60 min. The sets of sensor-measured and self-reported contacts had a large intersection, but neither was a subset of the other. Participants’ aggregated contact duration was mostly substantially longer in the diary data than in the sensor data. Twenty percent of respondents (>1 reported contact) stated that filling in the diary was too much work, 25 % of respondents reported difficulties in remembering contacts, and 93 % were comfortable having their conference contacts measured by sensors.

**Conclusion:**

Reporting and recording were not complete; reporting was particularly incomplete for contacts <5 min. The types of contact that both methods are capable of detecting are partly different. Participants appear to have overestimated the duration of their contacts. Conducting a study with diaries or wearable sensors was acceptable to and mostly easily done by participants. Both methods can be applied meaningfully if their specific limitations are considered and incompleteness is accounted for.

**Electronic supplementary material:**

The online version of this article (doi:10.1186/s12879-016-1676-y) contains supplementary material, which is available to authorized users.

## Background

Contact patterns are important drivers of infectious disease dynamics [1], and failure to incorporate relevant properties of network structures may result in faulty predictions of disease spread [2, 3] and, hence, inefficient allocation of scarce public health resources. Various methods have been developed to measure social mixing [4], in particular contact diaries [5–8], wearable sensors [9–16], and video analyses [17].

These methods are heavily used to parameterize models that inform public health policy [7, 18] and support contact tracing [19]. However our understanding lacks evidence regardingwhat kind of contact definitions are good proxies for situations when transmission happens, how well the existing methods capture these contacts [19–21], and how measurement quality differs with context. Among the few studies that addressed methodological aspects of contact measurements, five focussed on reporting problems in diary studies [20–24], two compared sensor-measured to diary-reported contact data [20, 21], both in a high school context, and one related sensor-measurements to direct observations [25]. Notably, some robust features have emerged from these studies: in particular, that reporting of transient contacts is poor whereas extended contacts are reported reliably [20–22]. Other results, such as reporting probabilities, vary between studies. Overall, more studies are needed, especially for contacts occurring in different contexts.

An important factor affecting the validity of contact measurements and the participants’ response in contact studies is acceptability, not only of the data collection as such, but also of the specific measurement method used. To our knowledge, there are no specific data available on the acceptability of wearable sensors used in contact studies, and only two studies assessed the acceptability of paper diaries [5, 8]. Both studies report positive subjective assessments, e.g., that filling in diaries seems easy to many participants. This is in contrast to the reporting errors observed, e.g., by Smieszek et al. [20, 22]. Moreover, both Smieszek et al. [20] and Mastrandrea et al. [21] obtained a higher participation rate for sensor-based measurements than for diaries collected in the same populations (high school members), i.e., many more students agreed to wear sensors and actually wore them than students were willing to complete and submit a contact diary.

Here, we aimed at a joint investigation of measurement errors in contact studies and participants’ attitudes and self-assessments. This paper contributes to the existing literature in three ways: First, we measured contacts with both contact diaries and wearable proximity sensors at a scientific conference in Germany, adding another setting to the few existing datasets that allow an analysis of measurement differences and errors. Second, we investigate the potential impact of these differences on estimates of the basic reproduction number, *R*_0_, and contact duration. Third, we investigate the sentiments of participants towards both methods, in particular regarding practicability, privacy, participation’s effects on social interaction, and the feasibility of potential future study designs.

## Methods

### Protocols, material, code and data

Code, questionnaire, contact diary as well as the final anonymous datasets are provided as online supplementary material (Additional files 1, 2, 3, 4, 5, 6, 7, 8, 9, 10, 11 and 12).

### Recruitment

We conducted a study involving participants of a scientific conference on epidemiology (“Jahrestagung der Deutschen Gesellschaft für Epidemiologie”, http://dgepi.de/jahrestagungen.html) in Ulm, Germany, September 2014. Study participants were recruited during the first half of the first conference day. Of the approximately 300 conference attendees, 100 could have participated in our study (number of available sensors); we were able to recruit 76 participants. All but one of them returned the sensors, and 74 contact diaries were handed back. Of these, 6 did not fill in any of the questions on acceptability and study design that were placed at the end of the diary booklet, and an additional 6 participants completed only the last page with questions on future studies, presumably by mistake.

### Data collection

#### Wearable sensors

We used active Radio Frequency Identification (RFID) sensors developed by the SocioPatterns collaboration; a precise description of the devices’ functionality has been published [15], which we briefly summarise here: Participants wore the sensors in a pouch that was attached to a lanyard; the sensors exchange low-power radio signals only on short distance (~1.5 m) if unobstructed [15], e.g., not covered by hands, and can thereby detect close-proximity face-to-face events between participants; detected interaction events are sent to and logged by radio receivers; the final data is aggregated with a temporal resolution of 20 seconds. Of note, it is possible that situations occur where two individuals are in close proximity but do not face each other, e.g., sitting next to each other in a lecture hall, resulting potentially in no registered contact event by the sensors while exchanging more than 10 words would be reported in the diary (see also paragraph “Difference in the contact definition” in the discussion). Fourteen receivers were deployed at the conference venue as depicted in Additional file 13: Figure S1. Activated sensors were given to the participants together with a contact diary after signing the consent form.

#### Paper-based contact diaries

We handed out paper-based contact diaries, which were similar in content to those from previous contact studies [6, 7, 22]. Participants were asked to report any contact with another study participant that fulfilled at least one of the two following conditions: (i) physical contact and/or (ii) mutual conversation of at least 10 words. Here, a contact with a specific person is defined as the aggregate of all individual contact events with that person throughout the entire study period. We equipped the pouches containing the sensors with clearly visible, short identifiers (IDs, consisting of a letter and a one-digit number, e.g., “B4”), which allowed study participants to recognise each other and to report contacts unambiguously. If participants were not able to recall the ID, they were still asked to report the contact and to tick an “ID unknown” box instead. Participants further logged for each contact gender and estimated age of their counterpart, an estimate of the aggregated (i.e., total) contact duration (categories: <5 min, 5-15 min, 15-60 min, and >60 min), kind of contact (physical contact and/or conversation), and familiarity with the counterpart (well-known or not).

#### Questionnaire on acceptability

A questionnaire on demographic data (gender and age in 10-year categories) and acceptability of the two methods was also part of the diary booklet. We asked participants to rate twelve acceptability items (5 categories, for details see Fig. 3 and Additional file 1), to estimate the time needed for completing the diary, to evaluate if study participation affected their contact patterns, and to assess their own, perceived, reporting quality. Finally, we asked the participants if they deemed potential long-term population-based study designs based on wearable sensors feasible.

### Data handling and processing

#### Booklet data entry, cleaning, and imputation

Diaries and questionnaires were entered twice by different staff members, and differences were resolved with the help of a third staff member. Missing information about a participant’s age or gender was inferred by using the age and gender information that was reported by the participants’ contact partners. Unambiguous imputation was possible for all missing gender entries and for all but one missing age entries.

#### Processing and cleaning of the sensor data

Raw contact data were retrieved from the 14 receivers, and processed following previously used protocols [15]. During the recruitment and deployment phase, sensors were successively prepared and activated by inserting the battery. However, deployment logistics required to prepare several sensors at once resulting in small heaps of activated sensors, which might have led to illegitimate contact recordings before or while the sensors were handed over to the participants. To correct this, we tested various filters and identified two, which produced contact data that were plausible and maximally consistent with the data from the diaries: (i) filtering out all groups of more than four simultaneous contact partners (filter 1), (ii) for filter 2, we proceeded in the same way, and additionally removed all contacts that involved already removed sensors and occurred immediately before or after the grouped contacts removed by filter 1. Filter 2 allowed us to discard sets of sensors laying activated on the table in changing constellations. Finally, the temporally resolved data were aggregated over the entire contact day.

#### Data matching

Diary and sensor data were matched directly when participants reported contacts including their counterpart’s ID. Contact reports with a missing or unknown ID were, for sensitivity analysis, treated as discordant or matched based on plausibility. In the latter case, we created two different matched datasets, one optimizing the concordance within the diary data, the other optimizing the concordance between sensor and diary data. A necessary condition for a plausible match was in either case that a potential counterpart matches the reported demographics. For the diary-optimized dataset, a second condition for a match was that the potential counterpart reported contact to the reporting participant; for the other dataset, the second condition was that the sensors recorded contact between the reporting participant and the potential counterpart. Contact reports with an “unknown ID” that could not be matched were deemed to represent discordant contact reports.

For reasons of data protection, we merged categories such that no demographic group (gender and age combined) had ≤5 participants; as a consequence, age has been dichotomised (<40 years, ≥40 years).

#### Resulting datasets

For both self-reported and sensor-recorded data, three datasets were available for analyses: The self-reported sets consist of (i) raw data, (ii) data with matched contact reports (diary-optimized), and (iii) matched contact reports (sensor-optimized). The sensor-recorded sets consist of raw data and filtered data, using filter 1 and 2. As results for both filtered datasets were similar, we only show results for the data obtained after applying the stricter filter, i.e., filter 2, and refer to them as filtered data.

### Analysis

#### Probabilities of reporting contacts

Our study population was a clearly defined group and, in an ideal world with perfect reporting, every contact among participants would be reported by both involved participants. Deviations from this ideal indicate reporting errors. To account for these, we estimated the complementary contact reporting probabilities using a unit square approach as described previously [20, 22] and validated with another method [26]. This approach is similar to capture-recapture methods [27]. We estimated the probability *P* of reporting a contact of specific duration, which is equivalent to sensitivity, based on the numbers of concordant and discordant contact reports under the following assumptions: (i) contact underreporting is possible, but no contacts are fabricated; (ii) contact reports are stochastically independent; (iii) in a matching pair of contact reports, we assume the higher duration value to be true if the reported durations are not equal. We approximated 95 % confidence intervals (CI) based on 10000 resamples of the study population (bootstrapping), and we also tested reporting differences between duration categories for statistical significance with these resamples.

#### Differences between diary and sensor data

The mapping of contact reports on sensor measurements allows estimating the probabilities (a) of a sensor-detected contact to be reported by a participant and (b) of a contact report to be also picked up by a sensor. The best estimates for these probabilities are the empirical proportions of reported or measured contacts. CIs and statistical significance were, again, determined using bootstrapping with 10000 resamples.

In addition, to estimate correlation between the numbers of distinct contact partners (degree) measured with both methods we computed Kendall’s τ-b and visualised the agreement of both methods with Bland-Altman-Plots.

#### Degree distributions and R_0_

We compared descriptively the degree distributions in the diary data, amended diary data (discordant reports made concordant by imputing unreciprocated reports), and in the sensor data. Mean degrees play a pivotal role in models of infection spread and for estimating the basic reproduction number *R*_0_. It is, however, known that both degree dispersion as well as contact durations and their distributions affect *R*_0_ [6, 28, 29]. Therefore, we report the results for the most basic correction, *R*_0,het_ = *R*_0,hom_∙(1 + CV^2^) - with CV being the coefficient of variation of the degree and the indices *hom* and *het* indicating *R*_0_ assuming a homogeneous degree and *R*_0_ corrected for degree heterogeneity, respectively [28, 29] - to determine how *R*_0_ differs from the homogeneous mixing model, when we allow for degree dispersion. We also report the results of the same correction for a degree information that only includes contacts of more than 15 minutes, as previous research suggested that these contacts might be more relevant for the transmission of several infections than shorter ones [30] and since contact duration is deemed to affect transmission probability [6]. Finally, we project relative differences in *R*_0,het_ between diary and sensor data (for both all contacts and >15 min contacts only), applying the common assumption in modelling that *R*_0,hom_ is proportional to the mean degree.

#### Differences in strength between diary and sensor data

We compared for each participant the strength [31], which is here the sum of the durations of all contacts a participant had with all its contacts partners throughout the study day, as measured by the sensors and as computed from contact diaries. The strength is considered a good indicator for a person’s potential for disease transmission [12, 32]. From the sensor data, we could derive strength point estimates. Duration in the self-reported data was a priori categorised. Hence, the diary strength of each participant was established as an interval ranging from the minimal to the maximal plausible strengths.

To compute the minimal plausible strength of an individual A, we consistently assume the smallest possible durations (including 0) for all contacts in which A was involved (i.e., either A reported a contact or another person reported a contact with A, or both). For the maximal plausible strength of an individual, we instead consistently used the upper bound of the reported categories for each contact in which that individual was involved. Since the highest duration category (>60 min) had no given upper bound, we defined an upper bound of two hours.

For statistical analyses, we also defined an intermediate strength, which was based on the mid-points of the duration categories. In a comparison of sensor-measured and diary-based strength values, we can distinguish three distinct cases: (i) participants whose diary-based strength range (minimum to maximum) enclosed their sensor-measured strength; (ii) those whose maximally plausible self-reported strength was less than their sensor-measured strength; (iii) those whose minimally plausible self-reported strength was higher than their sensor-detected one.

#### Questionnaire

For analysis of questionnaire items that required experience in keeping a contact diary (F1 to F4, Additional file 1), we considered only participants who reported contacts. To compare groups (e.g., differences between genders or age strata), Chi^2^- or Fisher’s Exact tests, when appropriate, were used for categorical variables and Wilcoxon or Kruskal-Wallis tests for continuous variables after evaluating distribution. We considered p-values < 0.05 as significant.

Analyses were conducted with STATA 12 IC (StataCorp LP) and, most of the contact data analyses, with code written for Enthought Canopy Python 2.7.3.

## Results

### Study population

Of 74 study participants, 61 % were female (*n* = 45) and 39 % were male (*n* = 29); 68 % were <40 years (*n* = 50) and 31 % were ≧40 (*n* = 23); for one person age was not reported. Of all participants <40 years, 74 % (*n* = 37) were female, whereas in the older age group 35 % (*n* = 8) were female.

### Description of contact data

In total, 329 contact reports were obtained from the contact diaries, corresponding to 199 contacts: 130 contacts were noted by both parties (corresponding to 260 reports), 35 contacts were only reported by one participant (incl. 3 contacts to a participant who did not return the contact diary), and 34 contacts with unknown ID were reported (Table 1). Thus, crude overall concordance was 66.3 % (130/196; denominator without the 3 contacts contacts to a participant who did not return the diary).

**Table 1:** Number of contacts stratified by duration as reported in the diaries

	Reported duration: higher value					
Reported duration: lower value	<5 min	5-15 min	15-60 min	>60 min	Duration missing	Σ
No report	**17** (20)	**4** (6)	**3** (4)	**0** (0)	**1** (2)	**25** (32)^a^
Unknown ID	**10** (14)	**8** (10)	**4** (4)	**4** (5)	**1** (1)	**27** (34)
<5 min	**30** (28)	**22** (19)	**3** (3)	**3** (3)	**n.d.**	**58** (53)
5-15 min	**n.d.**	**11** (11)	**8** (8)	**3** (3)	**n.d.**	**22** (22)
15-60 min	**n.d.**	**n.d.**	**17** (17)	**6** (5)	**n.d.**	**23** (22)
>60 min	**n.d.**	**n.d.**	**n.d.**	**22** (22)	**n.d.**	**22** (22)
Duration missing	**5** (5)	**5** (4)	**1** (1)	**0** (0)	**1** (1)	**12** (11)
**Σ**	**62** (67)	**50** (50)	**36** (37)	**38** (38)	**3** (4)	**189** (196)

For concordant reports that differ in duration, columns contain the higher, rows the lower duration report (i.e., there were 22 pairs of individuals such that one of the pair reported a contact of less than 5 min while the other one reported a contact of 5-15 min); discordant reports and unknown IDs are also shown in rows; bold numbers show data after matching (diary-optimized), numbers in parentheses show crude data; n.d. = not defined.

^a^Three contacts to a participant who did not return the diary are excluded from analysis.

After matching contact reports with unknown IDs with unreciprocated reports (diary-optimized matching variant), we obtained 189 contacts of which 137 were concordant (i.e., 137 pairs of individuals reporting each a contact with the other), 25 contacts that were only reported by one participant, and 27 remaining contacts with unknown ID. Overall, 54.5 % (103/189) of contacts were physical and 67.7 % (128/189) of contacts were with a well-known counterpart (see Additional file 13: Table S1 and Additional file 13: Table S2).

The sensors recorded 316 contacts among the study participants, which would have resulted in 632 contact reports in the diaries if both capturing systems had assessed all contacts completely (Table 2).

**Table 2:** Number of diary reports versus sensor recordings stratified by contact duration

	Sensor						
Diary	No detection	<5 min	5-15 min	15-60 min	>60 min	Unknown ID	Σ
No report	**n.d.**	**302** ^a^ (394^b^)	**9** (11)	**2** (2)	**0** (0)	**n.d.**	**313** (407)
<5 min	**49** (49)	**63** (52)	**5** (4)	**1** (1)	**0** (0)	**2** (14)	**120** (120)
5-15 min	**29** (27)	**37** (29)	**5** (5)	**2** (2)	**0** (0)	**0** (10)	**73** (73)
15-60 min	**18** (18)	**27** (26)	**6** (5)	**6** (6)	**0** (0)	**2** (4)	**59** (59)
>60 min	**3** (3)	**20** (11)	**16** (17)	**21** (24)	**2** (2)	**0** (5)	**62** (62)
Duration missing	**7** (7)	**8** (7)	**0** (0)	**0** (0)	**0** (0)	**0** (1)	**15** (15)
Σ	**106** (104)	**457** (519)	**41** (42)	**32** (35)	**2** (2)	**4** (34)	**642** (736)

Columns contain sensor measurements, rows diary reports; bold numbers show data after filtering (sensor data) and matching (sensor-optimized), numbers in parentheses show crude data; n.d. = not defined; ^a^272 ≦1 min duration, ^b^307 ≦1 min duration, both sensor-measured

Comparing the crude survey and sensor datasets, we found 191 contact reports in the diaries (out of 329) that were also recorded by the sensors; 407 instances where the participants did not report a contact, but a contact was recorded by the sensors; 104 diary contact reports that did not match with the sensor recordings and 34 had an unknown ID. However, comparing edited survey data including matched “unknown IDs” (sensor-optimized) with filtered sensor data, we observed 219 concordant measurements, 313 instances without a diary report that were sensor-recorded, 106 reports that were not measured by the sensors, and 4 reports with a remaining unknown ID (Table 2). Of the 313 instances that were only recorded by sensors, 87 % (272/313) had a duration of one minute or less, and 10 % (30/313) had a duration between one and five minutes. Hence, only 3 % (11/313) of the instances that were only recorded by the sensors had a duration of more than 5 minutes.

### Diary reporting probability

From the diary data, we estimated the probabilities that a contact according to the diary contact definition was reported for the four different duration categories. For the matched dataset (diary-optimized), we estimated *P* = 72.2 % [63.5-81.1 %] for durations <5 min, *P* = 86.4 % [77.3-94.3 %] for durations 5-15 min, *P* = 89.2 % [80.6-96.3 %] for durations 15-60 min, and *P* = 94.4 % [88.5-98.8 %] for durations >60 min. The estimated probability for durations <5 min was significantly lower than the estimates for all three other categories (*p* < 0.01); none of the other differences was statistically significant.

### Differences between sensor and diary data

For the filtered and matched datasets, we estimated a congruency of 33.9 % [28.0-40.5 %] for <5 min; 78.0 % [64.7-90.9 %] for 5-15 min; 93.8 % [84.4-100.0 %] for 15-60 min. The estimated probabilities for all duration categories were significantly different from all other estimates (each comparison *p* < 0.05). Most discrepancies in the shortest duration category (<5 min) were due to very short contacts (≦1 min). Contacts of ≦1 min duration had a congruency of 25.3 % [19.4-32.1 %], whereas all contacts of a longer duration within this category had a congruency estimate of >60 %.

We analysed if there was a potential gender or age effect in reporting of contacts <5 min (for details see Additional file 13); however, assessing the confidence intervals of the respective proportions, none of the group comparisons revealed a significant difference. Stratified estimates for the other duration categories were not meaningful because of very wide confidence intervals.

If using the reported contacts as denominator, and calculating the proportion of all reported contacts that were also detected by the sensor network, we estimated a detection probability 57.5 % [45.5-68.8 %] in the <5 min duration category; 60.3 % [46.0-75.0 %] in the 5-15 min category; 66.1 % [51.4-82.0 %] in the 15-60 min category; 95.2 % [87.0-100 %] in the >60 min category. The estimated probability for durations >60 min was significantly higher than the estimates for all three other categories (*p* < 0.01); none of the other comparisons was statistically significant. The detection probability of contact reports with missing duration was 53.3 % [20.8-87.5 %].

**Fig. 1:**
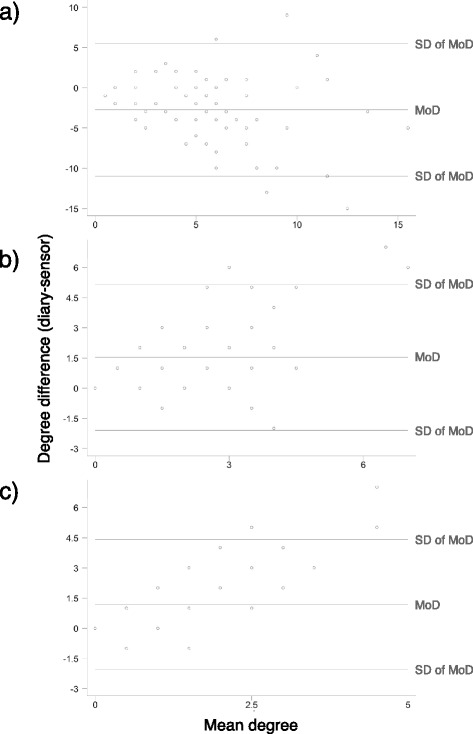
Bland-Altman plots for degree measured by both methods: including **a** all contacts, **b** only contacts **≥**5 minutes, **c ≥**15 minutes. SD: standard deviation, MoD: mean of degree difference

We further analysed if the reported and recorded degrees were correlated: Kendall’s τ-b as measure of degree correlation for both methods was for all contacts 0.29 (*p* <0.001), for contacts ≥5 min 0.50 (*p* <0.001), for contacts ≥15 min 0.37 (*p* < 0.001). Figure 1 illustrates differences in reported and recorded degree as a function of the mean degree of both measurements: For all contacts regardless of duration, diaries measure an average of 2.7 contacts per participant less than the electronic devices; for contacts ≥5 min they measure 1.5 contacts more and for contacts ≥15 min 1.2 more. All three constellations show very few outliers, with a systematic deviation to the effect that for both analyses with restriction on duration outliers occur only in one direction, i.e., that diaries measure more contacts (Fig. 1b and c).

### Degree distributions and reproduction numbers

Simple homogeneous mixing models typically define the basic reproduction number, *R*_0_, as the product of transmissibility, contact rate (here defined as mean degree per day), and duration of infectivity. However, *R*_0_ actually depends on a complex interplay between degree distribution [28, 29, 33, 34], contact duration and intensity distribution [6, 35], and biological factors [36]. Details of degree distribution parameters of our data are provided in Additional file 13: Table S3.

When calculating the basic reproduction number *R*_0_ accounting for degree heterogeneity, we found that one would expect the heterogeneous *R*_0.het_ to be between 133 % (recorded, filtered data) and 148 % (reported, crude data) of *R*_0,hom_ (when using the homogeneity assumption that all individuals have the same degree), and between 213 % (reported, matched) and 319 % (recorded, crude) when only considering contacts with a duration of at least 15 min (see Additional file 13: Table S3), which are thought to be of more relevance for the transmission of many infectious disease [30, 37, 38].

With *R*_0.hom_ being proportional to the mean degree and *R*_0.het_ being a function of *R*_0.hom_ and the coefficient of variation of the degree, we were able to project ratios of *R*_0.het_ and *R*_0.hom_ for various reported and recorded contact data from our study. When both short and long contacts are included in the analysis, the basic reproduction number *R*_0.het_ resulting from the diary data would only be 76 % of that coming from the sensor data. The opposite is true if only contacts >15 min are included in the analysis; then *R*_0.het_ from the reported data is 236 % of the corresponding value from the recorded data.

### Diary-reported versus sensor-detected strength

**Fig. 2:**
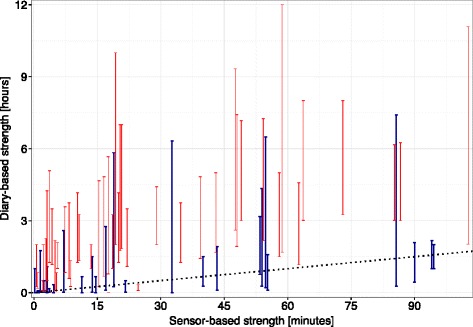
Sensor-based (abscissa) versus diary-based (ordinate) strength per participant. Diary-based strength is given as range, from the minimal plausible to the maximal plausible strength value that was consistent with the reported duration categories. Sensor-based strength values are point-estimates and jittered, where necessary. Dotted line indicates equal strength. Blue ranges include the corresponding sensor-based strength, red ranges exclude it

Figure 2 shows the plausible range of strength (aggregated duration of all contacts) values as obtained from diaries vs. the strength measured by sensors. In addition, Table 3 shows median strength in each of the comparison categories. More than one third of participants have diary-computed strengths compatible with sensor data, and diary data overestimate sensor data for more than half. An underestimation in the diaries with respect to sensors is obtained only for 6 participants.

**Table 3:** Diary- and sensor-based strength among the three groups that result from the diary-sensor-strength comparison

	Diary-based strength lower than the sensor measurement	Diary-based strength includes sensor measurement	Diary-based strength higher than the sensor measurement
N (%)	6 (8 %)	26 (35 %)	42 (57 %)
Diary-based strength (median)	0	43 min	171 min
Sensor-based strength (median)	3 min	18 min	19 min
Degree (median), diary	0^a^	4	5
Degree (median), sensor	3	8	6.5

^a^Only 1/6 participants reported contacts

### Self-assessed workload and reporting quality

The retrospectively assessed median time of filling in the contact diary was 5 minutes (interquartile range (IQR) 5-10, *n* = 61). It took participants 1.25 minutes per contact (median; IQR 1-2.5, *n* = 56) to fill in all required information. There was no statistically significant difference regarding gender (*p* = 0.45) or age (*p* = 0.42).

Seventy-eight percent (45/58, 95 %-CI 65-87) thought that they assessed the number of their contacts to other study participants correctly, i.e., that they did not forget or fabricate contacts; 21 % (12/58, 95 %-CI 11-33) thought they underestimated their number of contacts, and 2 % (1/58, 95 %-CI 0-9) reported an overestimation. We detected no statistically significant association of this self-assessment with gender (*p* = 0.38) or age (*p* = 0.33).

Using the absolute difference between self-reported number of contacts and sensor-measured ones as a marker for underreporting, respondents who classified themselves as “underreporter” had a median of 5 missing contacts (range 1-13) and respondents who assessed their reporting as correct had a median of 4 (range 0-15); no significant difference between these two groups was identified (*p* = 0.4).

Seventy-four percent (45/61, 95 %-CI 61-84) evaluated their social interaction as unchanged by the study (no statistical significant association with gender or age). Of those who perceived a change (16/61), 93 % (14/15, 95 %-CI 68-100) reported an increase in contact numbers and 90 % (9/10, 95 %-CI 55-100) thought their contacts were longer than without study participation.

### Acceptability

Only 20 % of respondents (11/54, 95 %-CI 11-34) with at least one reported contact stated that filling in the diary is too much work. A quarter of respondents reported difficulties in remembering contacts (13/53, 95 %-CI 14-38); however, 85 % of these (11/13, 95 %-CI 55-98) assessed themselves as having reported the right number of contacts. Reported easiness of filling in the diary, perceived workload and easiness of remembering contacts were not associated with the time spent per contact of filling in the diary (*p* = 0.72, *p* = 0.20 and *p* = 0.24) or number of contacts (*p* = 0.33, *p* = 0.33 and *p* = 0.51). Participants who rated filling in the diary and remembering contacts as easy reported a higher, but not significantly different, proportion of familiarity with their counterparts than those who disagreed: median of 60 % versus 50 % and of 62.5 % versus 50 %; however, participants with neutral positions had even higher median proportions (67 and 63.3 %).

**Fig. 3:**
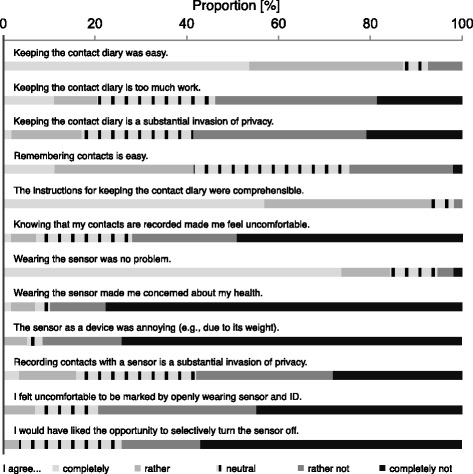
Answer categories to items of acceptability questionnaire (missing values: first two questions: *n* = 15; questions 3, 4, 5, 8, 9, 11, 12: *n* = 16; questions 6, 7, 10: *n* = 17)

Ninety-three percent (53/57, 95 %-CI 83-98) had no negative sentiment towards having their conference contacts measured by a wearable sensor. Additionally, only 16 % (9/57, 95 %-CI 7-28) see it as a strong invasion of privacy. Fig. 3 depicts the answer categories for all twelve items. We detected no significant association with age or gender for any of the items.

#### Future study design

When asked whether participants thought participation in a future study using wearable sensors would be possible for them if the device were to be worn once a week on a randomly chosen day over a one-year period, 51 % (28/55, 95 %-CI 37-65) agreed. Seventeen percent of respondents (10/59, 95 %-CI 8-29) agreed completely that a population-based study using wearable sensors or another digital detector is feasible, 69 % agreed rather or partly (41/59, 95 %-CI 56-81), and 14 % (8/59, 95 %-CI 6-25) dismissed the idea.

## Discussion

Both sensor-measured contacts and self-reported contacts were distinct sets with a large intersection, but none was an entire subset of the other. Using the diary reports, we estimated reporting probabilities between 72 and 94 %, with lower probabilities for shorter contacts, consistent with previous studies [20–22]. When comparing both contact measurement methods, we obtained a substantial overlap in reporting and recording, but also important differences, with in each case contacts reported or recorded by one method but not the other. Relating diary reports to sensor recordings, we found reporting probabilities between 34 and 94 %, again with lower probabilities for shorter contacts, and relating sensor recordings to diary reports, we estimated recording probabilities between 57 and 95 %. Both methods of contact measurement were acceptable to the participants in our specific setting: Only 20 % of respondents with at least one reported contact stated that filling in the diary was too much work and 25 % reported difficulties in remembering contacts. Almost all participants (93 %) were comfortable having their conference contacts measured by a wearable sensor.

### Differences between diary and sensor data

Differences between diary and sensor contact data can be classified into three different categories: (i) legitimate differences due to different underlying contact definitions, (ii) differences due to limited receiver coverage, (iii) differences due to reporting and measurement errors.

#### Differences in contact definitions

The proxy for a potentially contagious contact in contact diaries is social interaction, i.e., conversation and/or physical contact. The definition underlying sensor measurements is broader in a sense that every short-distance, face-to-face collocation of two participants was recorded. It is likely that in our study sensor-recorded face-to-face events without conversation or physical contact occurred that were legitimately not reported in the diaries. Conversely, participants might have reported conversations that were not clearly face-to-face but angled or even side-to-side, or over a distance of more than ~1.5 m, which were then not recorded by the sensors.

#### Under- and overrecording with sensors

The SocioPatterns infrastructure used here required stationary receivers to store contact data (unlike, e.g., TelosB motes [16], which store contact data directly on the sensors). As a consequence, only contacts that took place in an area covered by at least one receiver were recorded. The positioning of receivers is often - and was in our study - constrained by health-and-safety requirements (e.g., no exposed wires someone could trip over), the availability of power sockets, the arrangement and orientation of walls, etc. Blind spots in the study area due to these constraints might have been present. Furthermore, we only covered the rooms, lecture theatres, and open spaces used for the conference, but we did not cover the canteen or outdoor spaces (see Additional file 13: Figure S1). It is highly likely that some contacts took place there, too.

Underrecording can also happen when participants do not adhere to the instructions and place their sensor under a jacket or at another inappropriate place that shields electromagnetic signals. We observed, e.g., one participant placing his sensor in a backpack. These reasons as well as reporting of angled or distant conversations might explain why about 42 % of short contacts (less than 5 min) that were reported were not recorded by the sensors.

Overrecording happened during the recruitment phase, when activated sensors had to be distributed to many participants within very short time. We attempted to remove these artefactual recordings by filtering the raw data (see section “Processing and cleaning of the sensor data”).

Taken together, this highlights the fact that using sensors for contact measurements is not fully free of measurement errors.

#### Under- and overreporting with contact diaries

It is generally assumed that discordant reporting is due to underreporting and that no contacts are fabricated [20, 22, 26]. As expected, we found differences in reporting quality depending on duration of contact, with short contacts showing relatively more discordant reports than extended ones.

Our estimates of reporting probabilities were based on three assumptions, one of which was the independence of reporting. Unlike in previous studies, where the reporting was retrospective [20, 21] or well-integrated into the daily routine [22], participants in this study were branded by wearing the sensor pouch with their visible ID number and the study participation itself could have triggered conversation among participants. Hence, it is likely that the independence assumption was partly violated and concordant reports (in either way: both reporting or both not reporting) might have been overrepresented. As a consequence, true reporting probabilities might be lower than estimated here.

Of note, our results confirm two important facts: on the one hand, participants forget to report a proportion of – especially short – contacts [20–22]; on the other hand, there seems to be a tendency to overestimate contact duration if contacts are reported (see Fig. 2, and Mastrandrea et al. [21]). Indeed, diary-based estimation of individual strength are larger than the corresponding measure based on sensor data for about 60 % of participants, even if very strict definitions concerning the reported strength are applied (see method section). A possible reason for this discrepancy in duration estimates could be that durations of contacts as measured by sensors might not meet assumptions of participants about contacts, i.e., they might take the whole duration of a meeting as a contact and not brief periods with face-to-face contacts, leading to overreporting of durations. Another reason could be due to underrecording by the sensors as discussed above. We suppose that the latter factor does not explain a major part of the observed difference. Diary-based strength smaller than sensor-based strength was obtained only for few participants; however, given 5/6 of these participants did not report any contact, they had no real opportunity to bias their contact duration.

The majority of participants, nearly 80 %, thought that their reporting concerning the number of contacts was correct. This self-evaluation confirms findings from a previous study: in McCaw et al. most participants of this three-day paper diary study thought that they got the number of contacts right. [5] However, taking actual reporting errors into account, the comparison shows that even scientifically trained participants are subject to misperceptions (72 % reporting probability for short contacts).

A problem for contact studies is that measuring behaviour can influence it: about one quarter of our study participants reported more frequent and longer contact events that they attributed to the study, although in longer studies, e.g., over a week, this effect could wear off and it might be of less impact in studies without visible ID.

### Comparison of contact reporting with previous studies

The crude overall concordance of contact reporting, i.e., contacts that were reported by both parties, we observed in our study was at 68.8 % considerably higher than in previous studies conducted at a university (30.2 %), a US high school (23.5 %), and a French high school (44.9 %) [4, 20, 21] and similar to the one of a study done at a research institute over one week (65.0 %) [22]. Interestingly, Conlan et al. reported a concordance of 61 % from a contact study investigating social network primary school students aged 4 to 11 years [23]; however, in this study contact networks were built on general reports from children about their social network and were not specific to a given study period. Also, degree was a priori limited by questionnaire design. Both characteristics might contribute to high mutual reporting and render direct comparison with our study not feasible.

Stratifying for duration, the reporting probabilities in our study are qualitatively in line with previous studies. However, in Smieszek et al. [22], participants reported short contacts with a probability of 49.0 % compared to 72.2 % in our study, despite having a high reporting probability in other duration categories. One reason might be that the 2012 study asked the participants to report contacts over several days, which increases the overall workload of participants, and that there were more contacts to be reported. In Smieszek et al. 2014 [20], the reporting probabilities of all duration categories were considerably lower - with the exception of contacts >60 min - which might be due to the retrospective study design, and the particular group of study participants (high school students). Finally, Mastrandrea et al. [21] found 40 % reporting probability for contacts <5 min, and 72 % for contacts >60 min.

Reasons for the particularly high reporting probabilities in our study compared to previous ones could be: the low density of study participants among conference attendees and the fact that a very structured conference day is associated with a low workload; the study duration was also comparatively short (4 to 8 hours); moreover, the study population consisted mostly of scientists, who are familiar with filling in questionnaires and who are probably intrinsically motivated to produce data of good quality.

Interestingly, in our study the reporting by men was not lower than that of women, in contrast to findings from a previous study [20] where the reporting by female study participants was about twice as high as that of males. However, in that previous study, gender specific results might be specific to the age of most of the participants (teenagers) and hence not necessarily generalizable.

Comparing the correlations between reported and recorded degree with the results from Smieszek et al. 2014 [20], again, we found better correspondence between the data from both methods (Smieszek et al. 2014: τ = 0.01 (all contacts), τ = 0.14 (contacts ≥5 min), τ = 0.21 (contacts ≥15 min) with corresponding values of 0.29, 0.50 and 0.37. Nonetheless, the correlation between the degrees reported and recorded are still weak for all three combinations of contact duration.

### Implications of study findings for modelling

We found, in line with previous research, that the discrepancies within survey data as well as between sensor- and survey-data are highest in contacts of short duration. Several studies found that only mixing matrices of contacts ≥15 min could explain population-wide seroconversion of a multitude of infections better than, e.g., random mixing models [30, 37, 38]. If only contacts of a minimal duration of 15 min are included in infection transmission models, then the absolute number of discrepancies decreases substantially. If we believe short contacts not to be relevant then the high proportion of reporting errors in contacts of short duration might be not as problematic as it appears. However, all published evidence is based on self-reported contact data; it is very likely that the reporting of short contacts in those studies was also poor, which implies that the findings might be biased.

Even though non-intense and short contacts most likely play a role in infection transmission, the transmission probability per contact for such contacts can be expected to be substantially lower than for extended contacts for most infectious diseases [6, 30, 32, 37–39], which is – besides perhaps poor data quality – most likely one of the reasons why no study has found short contacts to be explanatory for serological survey data.

We also found that the correction for pure degree heterogeneity results in higher *R*_0_ increases with respect to a homogeneous mixing *R*_0_ hypothesis for contacts ≥15 min than for all (short and long) contacts: in other words, contact data including short and long duration contacts were found to behave closer to random mixing [16, 36] than networks containing only contacts of longer duration, which are much sparser and more structured. Of note, our diary data are expected to produce lower *R*_0_ values than the sensor data (for identical transmissibility and infection duration) if all contacts are considered, but much higher *R*_0_ for contacts ≥15 min only. The underlying reason for this, i.e., the situation that shorter contacts are reported less and longer contacts are reported slightly more often than recorded, is also reflected in the Bland-Altman plots (Fig. 1) and may be due to the fact that the durations of contacts were estimated to be longer than the corresponding recording of duration by sensors, as discussed above.

### Acceptability

When interpreting participants’ attitudes and assessments we have to bear in mind the specific setting the study took place in, i.e., a semi-public space where aspects of privacy, social control and familiarity with other people might be different both from completely public or private spaces. Privacy regarding both reporting contacts in a diary and being recorded with a digital device worried only a minority of respondents in our setting. The same applies for being tagged with the visible ID. Consistent with this, only a very small minority of participants would like to be able to turn off the sensor. In all, respondents had a very relaxed attitude towards reporting and being reported in our specific setting. Apart from aspects of social control (that are already in place at a conference setting and cause contact measurements not to be an additional control), it might be the case that our participants – mostly German epidemiologists – know and trust the national data protection laws. Hence, issues of privacy might play a more prominent role in different study settings or populations.

In planning future studies, one should take into account that only ca. 50 % of respondents said they would participate in a year-long study with weekly recording for 1 day over the period of one year. This leads to the assumption that network studies can be conducted successfully only over a couple of weeks and/or in social contexts with a strong social cohesion (which might limit generalizability) or need to provide an effective way to motivate potential participants [21, 23, 40]. Unfortunately, social dynamics can also play a negative role and may decrease compliance [20]. Thus, it might be difficult to recruit people into long-term contact studies; however, a majority of those who participated did not object to using diaries or sensors and expressed mostly neutral to positive sentiments. Furthermore, we might want to consider when planning future studies that about 20 % of the participants thought that filling in the diary was too much work; this was also the main concern of non-participants (data not shown). This sentiment leads to the hypothesis that contact studies using digital devices would have a higher response proportion than diary-based ones, as indeed observed in [21].

Compared to literature on attitudes towards contact diaries, our findings are similar. In the study by Beutels et al. about two-thirds of the participants of a two-day paper-based contact diary study associated “little effort” with the diary and in McCaw et al. nearly two-thirds of the participants rated filling in a diary as easy [5, 8]: in our study, nearly 90 % evaluated filling in the diary as easy. In addition, one other issue has been already addressed by a previous study: in Beutels et al. about 60 % of study participants saw (nearly) no change of number or kind of contacts by keeping the diary [8]; in our study this corresponds to 74 % with the lower limit of the confidence intervall (61 %) conveying no relevant departure from the previous finding.

### Limitations

Wearable sensors record contacts only among people that are equipped with them, meaning that only contacts within clearly defined groups can be measured. While diaries can be distributed to, e.g., a random sample of any population [7], any approach for assessing reporting errors from diary data also requires clearly defined participant groups. This necessary restriction limits the generalizability of studies’ results: Different groups most likely vary in cognitive capability or motivation. Furthermore, the difficulty of the reporting task differs between study settings. Our study had a low participant density (about 30 % of all conference participants and a median degree of 4) so that the corresponding low workload might have resulted in fewer errors. Also, some items of the acceptability survey might be positively influenced by the low workload, e.g., easiness of filling in the diary or remembering contacts.

Further, it is likely that self-selection biased responses on acceptability towards more positive answers: conference attendees who *a priori* deemed the study too demanding or unappealing were probably more likely to refuse participation than others with positive sentiments towards the study. Selection bias might not only have influenced study results by deterring conference attendees with a high expected number of contact events, but also shy attendees or people who are unfamiliar with the community. Whereas the first might lead to an underestimation of reporting errors, the latter might result in the opposite effect. Finally, regarding statistical differences between or among groups, our study might have not enough power to detect existing differences.

## Conclusion

Using data from contact diaries for analysing and explaining the transmission of highly contagious infections, i.e., when even short and non-intensive contact may be sufficient for transmission like it might be the case with, e.g., pertussis, is questionable. Studies conducted in three different settings (scientific conference, high school, workplace) have independently shown that reporting of short contacts (<15 min) is unreliable [20–22]. Reporting of extended contacts is almost complete, but missing data might also not be at random (since, e.g., participants certainly also differ in their levels of motivation for reporting contacts correctly).

Conducting a contact study with diaries or wearable sensors within the context of a scientific conference is well accepted and mostly easily done by participants. However, we have to recognise that measuring contacts might modify them in number and duration, especially if the study involves wearing visible IDs. This effect could be of less importance for studies that carry on for several days and could increase if participants have to report (or are recorded by digital devices) contacts that occur in social situations less desirable by society than a scientific conference.

There are studies that showed a relationship between contact patterns and patterns of infection for both wearable sensors and contact diaries [30, 41, 42]. However, another study did not find that empirical contact matrices explained seroepidemiological data of pertussis transmission better than, e.g., homogeneous mixing [43]. Since contact data are used to inform public health policy, it is pivotal to deepen our understanding of which events actually result in infection transmission and which contact proxies capture those events best. Three types of studies would be worthwhile for future research: (i) a comparison between contact diaries, wireless sensor networks, and video-assisted observations at the same time, to achieve method triangulation; (ii) modelling studies that test to what extent the presented differences in contact structure between the methods and measurement biases affect model outcomes; (iii) studies that relate transmission events based on pathogen detection to empirical contact data of the same setting to delineate which contact proxy works best and to improve our knowledge about disease transmission.

## Abbreviations

CI, confidence interval; CV, coefficient of variation; ID, identifier; IQR, interquartile range; RFID, radio frequency identification

## Additional files

Additional file 1: Study booklet. (PDF 187 kb) Additional file 2: Codebook. (XLSX 27 kb) Additional file 3: Acceptance data. (CSV 6 kb) Additional file 4: Contact data – sensors filtered. (CSV 5 kb) Additional file 5: Contact data – sensors filtered (extended). (CSV 5 kb) Additional file 6: Contact data – sensors unfiltered. (CSV 6 kb) Additional file 7: Contact data – survey matched to sensors. (CSV 5 kb) Additional file 8: Contact data – survey matched to surveys. (CSV 5 kb) Additional file 9: Contact data – survey. (CSV 5 kb) Additional file 10: Demographic data. (CSV 4 kb) Additional file 11: Contact analysis. (PY 67 kb) Additional file 12: Questionnaire analysis. (DO 10 kb) Additional file 13: Supplementary Figure and Tables. (PDF 304 kb)
