# Supplementary material for: Contact diaries versus wearable proximity sensors in measuring contact patterns at a conference: method comparison and participants’ attitudes
Source: BMC Infect Dis. 2016 Jul 22;16:341. doi: 10.1186/s12879-016-1676-y (PMC4957345; doi:10.1186/s12879-016-1676-y)
Supplement: Additional file 1: — Study booklet. (PDF 187 kb) [file 12879_2016_1676_MOESM1_ESM.pdf]

HELMHOLTZ
CENTRE FOR
INFECTION RESEARCH

Contact Diary

Variable name	Question	Answer categories	Coding
id	This contact diary belongs to ID	XX	new number
sex	Your gender	female	1
		male	2
age	Your age	younger than 30 years	1*
		30-39	2*
		40-49	3**
		50-59	4**
		60-69	5**
		older than 69 years	6**
* Recoded as 7.<br>** Recoded as 8.			

Definition of Contact

In this study, a contact is defined as a social interaction with another person who also takes part in the study. Participants can be recognized by their ID-tag and sensor. For a contact, at least one of the following two conditions is necessary:

(1) There was physical contact (e.g., shaking hands, backslapping, etc.).

(2) There was interactive conversation with, in total, at least ten words, spoken.

*NOTE: Please report **each contact partner only once** in your diary – even if you have contact with her/him several times. We are only interested in your estimate of the **aggregate duration** of contact with each person you meet today.*

*If you meet a person several times, please add up all durations of the individual contact events and mark a higher value accordingly. If you want to correct to a **lower value**, please make your intention clear (e.g., with an arrow). If marks are ambiguous, we will consider the highest marked value as valid.*

Please do not note any names!

Examples

During a conference break, Mrs Example meets Mr Bright. They shake hands and talk for slightly less than 15 minutes. Later on, they meet again, and Mrs Example introduces Ms Eager, her collaborator, to him. This conversation takes about 4 minutes. Mr Bright has ID "A6" and is about 60 years old. Ms Eager has ID "C9" and is 25 years old.

Mrs Example reports the contacts as follows:

ID: A6<br>o unknown ID	o female<br>x male	o < 30<br>o 30-39<br>o 40-49<br>o 50-59<br>x ≥ 60	o < 5 min<br>x 5-15 min<br>o 15-60 min<br>o > 60 min	x conversation<br>x physical contact	x yes<br>o no
ID: C9<br>o unknown ID	x female<br>o male	x < 30<br>o 30-39<br>o 40-49<br>o 50-59<br>o ≥ 60	x < 5 min<br>o 5-15 min<br>o 15-60 min<br>o > 60 min	x conversation<br>o physical contact	x yes<br>o no

Later on, Mrs Example talks with another scientist for about 10 minutes. This scientist was rather young; she estimates him to be in his mid-thirties; but she is not sure. Unfortunately, Mrs Example forgot to note the scientist's ID when she saw him, and she cannot recall it.

ID:<br><br><br><br>x unknown ID	o female<br>x male	o < 30<br>x 30-39<br>o 40-49<br>o 50-59<br>o ≥ 60	o < 5 min<br>x 5-15 min<br>o 15-60 min<br>o > 60 min	x conversation<br>o physical contact	o yes<br>x no

**ID**<br>*Please report the ID of the contact*	**Gender**<br>*Please report the gender of the contact*	**Age**<br>*Please estimate the age of the contact*	**Duration**<br>*Total duration of all contact-events*	**Kind**<br>*Please mark all kinds of contact that apply*	**Relationship**<br>*Do you know the contact person well?*
ID: XX<br><br>o unknown ID [88]<br><br>[missing data: 99]	o female [1]<br>o male [2]<br><br>[missing data: 9]	o < 30 [1*]<br>o 30-39 [2*]<br>o 40-49 [3**]<br>o 50-59 [4**]<br>o ≥ 60 [5**]<br>(missing data [9])	o < 5 min [1]<br>o 5-15 min [2]<br>o 15-60 min [3]<br>o > 60 min [4]<br>[missing data: 9]	o conversation [1]<br>o physical contact [2]<br>(both [3], missing data [9])	o yes [1]<br>o no [2]<br>(missing data [9])

Coding in [].
* Recoded as 7.
** Recoded as 8.

Final Questions
To learn from your experience, we would like you to evaluate the use of contact diary and sensor.

**Variable name**	**Question**	**Answer categories**	**Coding**
f1	Filling in the contact diary was easy.	I totally agree.	1
		I rather agree.	2
		neutral	3
		I rather disagree.	4
		I totally disagree.	5
f2	Filling in the contact diary is too much work.	as above	
f3	To register contacts with a contact diary is a strong invasion of privacy.	as above	
f4	Remembering contacts is easy.	as above	
f5	The instructions for filling in the contact diary were easy to understand.	as above	
f6	To know that my contacts are electronically registered made me feel uncomfortable.	as above	
f7	Wearing the sensor was no problem.	as above	
f8	Wearing the sensor made me be concerned about my health.	as above	
f9	The sensor as a device was disturbing (e.g. because of its weight).	as above	
f10	To electronically register contacts with a sensor is a strong invasion of privacy.	as above	
f11	It felt uncomfortable to be especially marked by openly wearing sensor and ID.	as above	
f12	I would have liked to be able to turn off the sensor intentionally.	as above	
time	How long did it take you to fill in the contact diary?	XXX minutes	-

Variable name	Question	Answer categories	Coding
quality	How well did you manage to estimate the number of your contacts? My estimation was	too low.	1
		correct.	2
		too high.	3
stb	Do you think the duration and number of contacts was different because of your participation in the study (compared to how they would have been without your study participation)?	No, my contacts did not differ.	0 [stb_i=88, stb_ii = 88]
		If not 0	1
stb_i	Yes, because of the study, my number of contacts was rather	lower	1 [stb = 1]
		higher	2 [stb = 1]
stb_ii	Yes, because of the study, my contacts were rather	shorter	1 [stb = 1]
		longer	2 [stb = 1]
longterm	From your experience, what do you think about the following idea for a study?	I can imagine wearing a sensor once a week (day chosen by chance) for a study that takes one year.	1
		I can**not** imagine to wear a sensor once a week (day chosen by chance) for a study that takes one year	0
longterm_day	Instead, I would possibly do that for a maximum duration of	XX days.	
longterm_week		XX weeks.	
longterm_month		XX months.	
feas	Do you agree to the following? A population based study, in which sensors or other digital positioning systems are used, is possible.	I totally agree.	1
		I rather agree.	2
		I partly agree.	3
		I rather disagree.	4
		I totally disagree.	5
