# Supplementary material for: Contact diaries versus wearable proximity sensors in measuring contact patterns at a conference: method comparison and participants’ attitudes
Source: BMC Infect Dis. 2016 Jul 22;16:341. doi: 10.1186/s12879-016-1676-y (PMC4957345; doi:10.1186/s12879-016-1676-y)
Supplement: Additional file 13: — Supplementary Figure and Tables. (PDF 304 kb) [file 12879_2016_1676_MOESM13_ESM.pdf]

Additional file 13

Supplementary Results, Figures and Tables

Supplement to: Timo Smieszek, Stefanie Castell, Alain Barrat, Ciro Cattuto, Peter J. White, Gérard Krause. Contact diaries versus wearable proximity sensors in measuring contact patterns at a conference: method comparison and participants' attitudes.

Supplementary Text

Gender and age effects in reporting of very short contacts

Of the sensor-recorded <5min contacts of participants younger than 40, 28.2% [21.9%-35.1%] were reported by them against 42.9% [32.4%-55.4%] of those of older participants. Analysis stratified by gender resulted in reporting probabilities of 31.2% [23.5%-40.4%] for <5min contacts in female and 37.8% [28.9%-46.9%] in male participants. Combining age and gender in the analysis, the following reporting probabilities for contacts in the <5min category were observed: young females 29.3% [22.2%-37.6%] vs. young males 25.3% [13.2%-39.1%], and older females 37.5% [16.7%-70.0%] vs. older males 46.4% [36.8%-55.2%].

Supplementary Figure

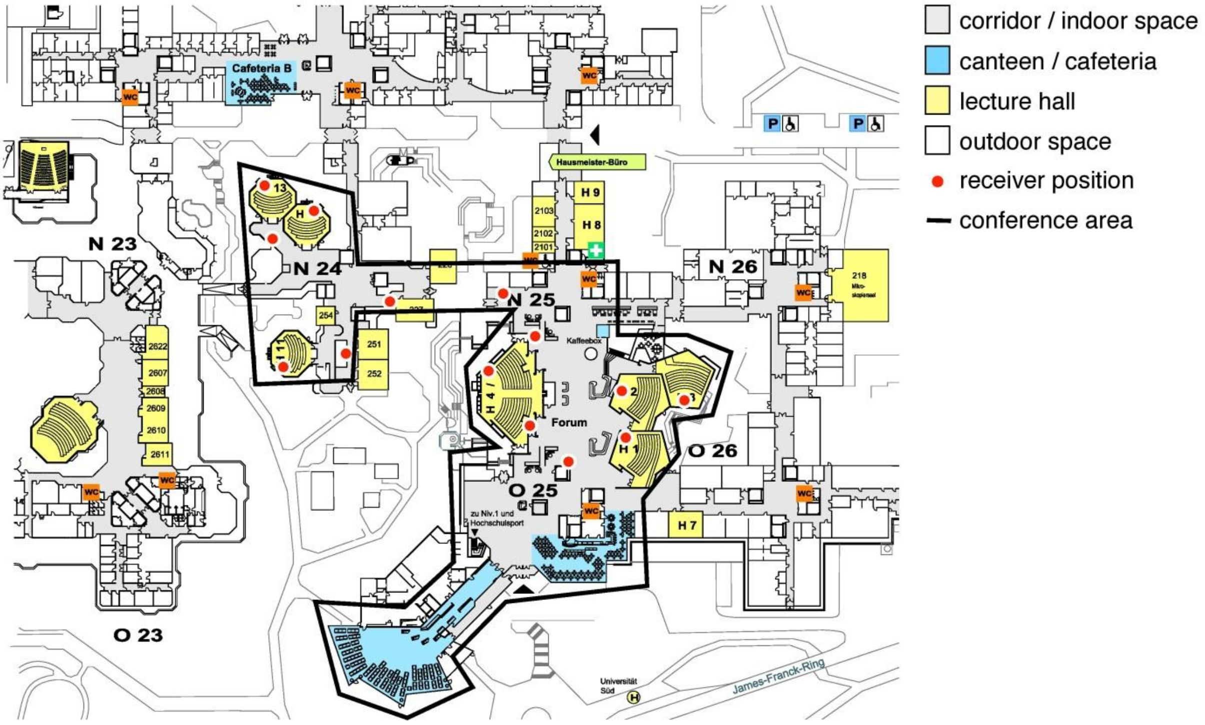

Figure S1: Floor plan of university campus where the conference took place. Conference sessions took place in lecture halls H1, H2, H3, H4/5, H11, H12, H13 (lecture halls with receivers). Red dots show receiver positions; the canteen could not be covered with

receivers. This figure is an adapted version of a floor plan that was kindly provided by “Vermögen und Bau Baden-Württemberg, Amt Ulm.”

Supplementary Tables

Table S1: Number of contacts stratified by kind of contact as reported in the diaries.

**Kind of contact: less intense**	**Kind of contact: more intense**				
	conversation	physical	both	missing	Σ
no report	**16** (20)	**5** (5)	**4** (7)	**0** (0)	**25** (32)
unknown ID	**19** (23)	**2** (2)	**5** (8)	**1** (1)	**27** (34)
conversation	**49** (46)	**6** (6)	**19** (17)	**0** (0)	**74** (69)
physical	**n.d.**	**3** (3)	**18** (18)	**0** (0)	**21** (21)
both	**n.d.**	**n.d.**	**37** (35)	**0** (0)	**37** (35)
missing	**1** (1)	**2** (2)	**2** (2)	**0** (0)	**5** (5)
Σ	**85** (90)	**18** (18)	**85** (87)	**1** (1)	**189** (196)

For concordant reports that differ in their classification, columns contain the more, rows the less intense category; discordant reports and unknown IDs are also shown in rows; bold numbers show data after matching (diary-optimized), numbers in parentheses show crude data; n.d. = not defined.

Table S2: Number of contacts stratified by how well the contact partner is known (as reported in the diaries).

**Reported familiarity: more known**	**Reported familiarity: less known**			
	known	unknown	missing	Σ
no report	**18** (23)	**7** (9)	**0** (0)	**25** (32)
unknown ID	**10** (16)	**17** (18)	**0** (0)	**27** (34)
known	**86** (81)	**11** (10)	**0** (0)	**97** (91)
unknown	**n.d.**	**35** (34)	**0** (0)	**35** (34)
missing	**3** (3)	**2** (2)	**0** (0)	**5** (5)
Σ	**117** (123)	**72** (73)	**0** (0)	**189** (196)

Bold numbers show data after matching (diary-optimized), numbers in parentheses show crude data; n.d. = not defined.

Table S3: Degree distributions and impact on basic reproduction number R_0.

Dataset	Duration included	*Mdn* (IQR)	*M* (*SD*)	Range	CV [%]	$R_{0,het}/R_{0,hom}$ [%]
Reported (crude)	All	4 (2-5)	4.4 (3.1)	0-14	69.6	148
	>15 min	1 (0-3)	1.6 (1.8)	0-8	109.0	219
Reported (matched and discordant missing contacts imputed)	All	5 (3-6)	5.1 (3.4)	0-15	65.5	143
	>15 min	1 (0-3)	1.7 (1.8)	0-8	106.2	213
Recorded (crude)	All	7 (4-10)	8.1 (4.9)	1-22	61.2	137
	>15 min	0 (0-1)	0.5 (0.7)	0-3	148.0	319
Recorded (filtered)	All	7 (4-9)	7.2 (4.1)	1-20	57.5	133
	>15 min	0 (0-1)	0.5 (0.7)	0-2	144.0	307

Reported contact data: N=74; recorded contact data: N=76; Mdn: median; IQR: interquartile range; M: mean; SD: standard deviation; CV: coefficient of variation; $R_{0,het}/R_{0,hom}$: ratio of R_0 corrected for degree heterogeneity and the uncorrected one.
